# Extension of chronological life span by reduced TOR signaling requires down-regulation of Sch9p and involves increased mitochondrial OXPHOS complex density

**DOI:** 10.18632/aging.100016

**Published:** 2009-01-28

**Authors:** Yong Pan, Gerald S. Shadel

**Affiliations:** ^1^ Department of Pathology, Yale University School of Medicine, New Haven CT 06520; ^2^ Department of Cell Biology, Yale University School of Medicine, New Haven CT 06520; ^3^ Department of Genetics, Yale University School of Medicine, New Haven CT 06520

**Keywords:** TOR signaling, TOR1, SCH9, mitochondria, proteomics, life span, aging, respiration, translation, ROS

## Abstract

The nutrient-sensing target of rapamycin (TOR) pathway appears to have a conserved role in regulating life span. This signaling network is complex, with many downstream physiological outputs, and thus the mechanisms underlying its age-related effects have not been elucidated fully.  We demonstrated previously that reduced TOR signaling (intor1Δ strains) extends yeast chronological life span (CLS) by increasing mitochondrial oxygen consumption, in part, by up-regulating translation of mtDNA-encoded oxidative phosphorylation (OXPHOS) subunits. Here, we have examined in greater detail how TOR signaling influences mitochondrial function and CLS and the role of the Sch9p kinase in the TOR-mitochondria pathway. As is the case for oxygen consumption, mitochondrial translation is elevated in tor1Δ strains only during active growth and early stationary phase growth points.  This is accompanied by a corresponding increase in the abundance of both mtDNA-encoded and nucleus-encoded OXPHOS subunits per mitochondrial mass.  However, this increased OXPHOS complex density is not associated with more mitochondria/cell or cellular ATP and leads to an overall decrease in membrane potential, suggesting that TOR signaling may influence respiration uncoupling. Finally, we document that the Sch9p kinase is a key downstream effector of OXPHOS, ROS and CLS in the TOR-mitochondria pathway.  Altogether, our results demonstrate that TOR signaling has a global role in regulating mitochondrial proteome dynamics and function that is important for its role in aging and provide compelling evidence for involvement of a "mitochondrial pre-conditioning" effect in CLS determination.

## Introduction

How and why we age has long been a fascination of humans.  In addition to being of intrinsic philosophical, evolutionary and biological interest, determining the molecular and cellular mechanisms underlying the aging process is relevant to understanding age-related pathology that ultimately limits human life and health span. Model organism studies have been instrumental in understanding aging,  with many  conserved pathways and factors having been identified in files, worms and yeast (and other organisms) that have physiological and pathological relevance in humans [1]. One general area that has been implicated strongly in aging and life span determination is nutrient availability/sensing. For example, dietary (i.e. caloric) restriction extends life span and ameliorates many of the age-associated declines in cellular function in virtually all organisms examined to date [2].

One major consequence of changing nutrient availability/sensing is alternation of cellular metabolism and mitochondrial respiration. Life span extension by caloric restriction, for instance, usually involves enhanced mitochondrial activity [2,3].  While best known for providing ATP via oxidative phosphorylation (OXPHOS), mitochondria are a major crossroads for anabolic and catabolic metabolism, as well as many other critical cellular functions such as apoptosis, signal transduction, and ion homeostasis [4].  Mitochondria also contain a DNA genome (mitochondrial DNA; mtDNA) that harbors a set of genes involved in OXPHOS and requires dedicated machinery for organellar DNA replication and gene expression that is encoded primarily by genes in the nucleus (e.g. mitochondrial DNA and RNA polymerase, ribosomes, transcription and translation factors, etc) [5,6].   Mitochondria also generate reactive oxygen species (ROS) as byproducts of the electron transport process, which is a major way they are thought to contribute to the aging process. For example, the "mitochondrial theory of aging", which builds on Harman's "free-radical" theory, posits that ROS from mitochondrial respiration damage cellular components, including mtDNA, and lead to declines in cell, tissue and organismal function over time [7,8].  As ROS are also signaling molecules, altered signal transduction is another potential contributor to aging phenotypes due to mitochondrial dysfunction [9]. While the mechanisms through which altered respiration affects life span are complex and have not been defined fully, differential ROS production is likely involved.  For example, aberrant respiration due to defective RAS signaling [10], pharmacological inhibition [11], or imbalanced translation of mtDNA-encoded OXPHOS subunits [9] elevates cellular ROS and severely curtails yeast chronological life span (CLS).  Conversely, mild uncoupling of mitochondrial respiration extends yeast CLS and decreases ROS [11].

Several kinase pathways serve as physiological switches in response to nutrient availability.  For example, the conserved target of rapamycin (TOR) signaling pathway controls growth by positively regulating the processes of ribosome biogenesis and cytoplasmic translation when preferred nutrient supplies are available.  In yeast, the TOR pathway also negatively regulates stress response genes, autophagy, and usage of alternate carbon and nitrogen sources [12]. Thus, when nutrients are limiting, TOR activity is reduced, energy is conserved (by shutting down expensive growth-promoting pathways) and diverted to provide stress resistance and access to alternate energy stores.  The TOR kinase forms two multi-protein complexes, TORC1 and TORC2, with TORC1 functioning as the nutrient sensor [12]. In yeast, there are two TOR kinase genes *TOR1* and *TOR2*.  Both Tor1p and Tor2p can function in the TORC1 complex, but only Tor2p can function in the TORC2 complex.  Thus, deletion of *TOR1* results in reduced TORC1 signaling, but is not lethal.  This is because Tor2p can partially cover the loss of Tor1p in TORC1, while still also functioning in TORC2.  In contrast, deletion of *TOR2* is lethal [13].  Reduced TORC1 signaling extends life span in a number of model organisms including yeast (*S. cerevisiae*), worms (*C. elegans*) and flies (*D. melanogaster*) [14-17].  We recently reported that a major mechanism underlying this phenotype in yeast is enhanced mitochondrial respiration driven, at least in part, by increased translation of mtDNA-encoded OXPHOS subunits [18]. In that study, we speculated that the extension of CLS by reduced TOR signaling involves an increase in the number of OXPHOS complexes per organelle that increases oxygen consumption, decreases ROS production in stationary phase, and thereby limits damage to cellular components.  However, since mtDNA encodes only minority of the OXPHOS complex subunits (i.e. of the ~80 OXPHOS subunits only seven in yeast and thirteen in mammals are encoded by mtDNA) and mitochondria contain >1,000 proteins (encoded by nuclear genes and imported into the organelle), the possibility that TOR signaling regulates mitochondria in a more global fashion is likely.  In fact, TOR-dependent changes in the mitochondrial proteome have been documented in human Jurkat T cells [19].

Sch9p belongs to the AGC family of kinases and is a key downstream target of TORC1 signaling in yeast.  For example, Sch9p is a functional ortholog of ribosomal protein S6 kinase, a key mediator of mTOR signaling in mammalian cells [20]. TORC1 directly phosphorylates Sch9p at multiple sites, which is important for modulating cytoplasmic translation and cell cycle progression.  Sch9p is also a negative regulator of both chronological and replicative aging [14,21] and has recently been shown to similarly regulate mitochondrial respiration [22].  In fact, like deletion of *TOR1*, deletion of *SCH9* extends yeast CLS in a respiration-dependent fashion, suggesting that Sch9p could be a downstream mediator of TOR-dependent mitochondrial OXPHOS regulation in this regard. In the current study, we have examined in greater mechanistic detail how the yeast TOR pathway influences mitochondrial gene expression, OXPHOS activity, and proteome composition, and the role of the Sch9p kinase as a downstream mediator of its effects on mitochondria.

## Results

### Reduced TOR signaling globally increases mitochondrial translation and results in a greater number of OXPHOS complexes per organelle

**Figure 1.**
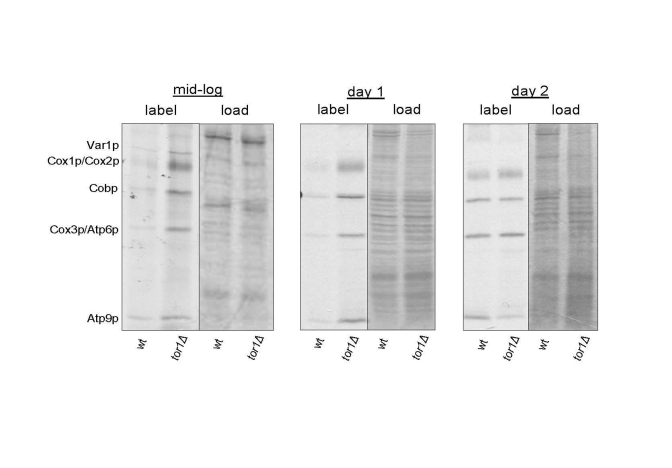
Elevated mitochondrial translation rates in tor1Δ strains during the exponential and early stationary growth phases. Results of an in vivo-labeling experiment in which the mtDNA-encoded gene products are labeled specifically and visualized by autoradiography after separation by SDS-PAGE (see Materials and Methods).  Wild-type (wt) and tor1 null (tor1Δ) strains labeled at mid-log, early stationary (day 1) and later stationary (day 2) are shown. The left-half panel under each time point is the autoradiogram showing the labeled mitochondrial gene products (with each product indicated on the left) and the right-hand panel is the respective Coomassie blue-stained gel as a control for total protein loading.

We demonstrated previously that reduced TOR signaling (in *tor1* null yeast strains; *tor1Δ*) results in increased mitochondrial translation rates, oxygen consumption, and life span [18]. This is accompanied by a corresponding increase in the steady-state levels of mtDNA-encoded OXPHOS subunits. However, whether there is global up-regulation of mitochondrial translation was not addressed in that study and only a single, late culture growth point was analyzed. To better understand the mitochondrial translation response to reduced TOR signaling, we labeled all mtDNA-encoded subunits at three growth points and visualized the individual products by autoradiography of SDS-PAGE gels. Compared to wild-type strains, we observed global up-regulation of mitochondrial translation products in log-phase and early stationary phase (day 1) cultures in *tor1D* strains (Figure 1). One day later in stationary phase (day 2) the wild-type and *tor1Δ* strains showed similar rates of mitochondrial translation, due to an increase in the rate in the wild-type strains (Figure 1). These results mirrored closely our previously published results on oxygen consumption as a function of growth state and demonstrate that the major differences in mitochondrial function in these strains are manifest during growth and early stationary phase, which is when TOR signaling is at its highest in wild-type strains.

**Figure 2.**
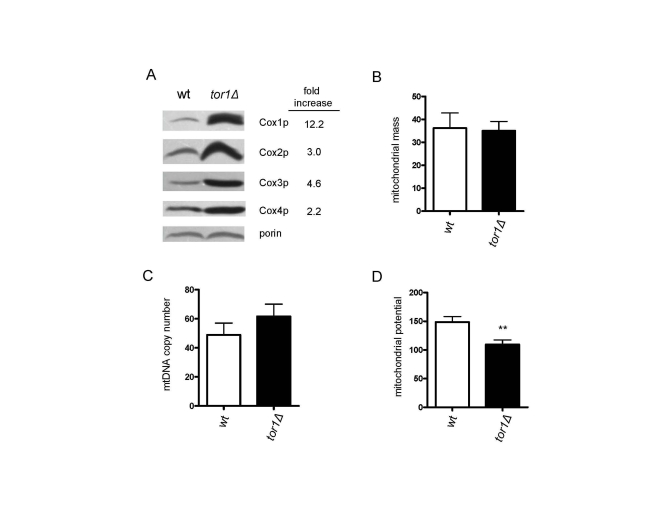
Reduced TOR signaling increases the number of mitochondrial OXPHOS complexes per organelle, as opposed to the number of mitochondria/cell. Comparative analysis of four mitochondria-related parameters in wild-type (wt) and *tor1Δ* strains is shown.  (**A**) Western blot analysis of four OXPHOS subunits (Cox1p-4p) and porin (as a mitochondrial normalization control).  Fifty μg of mitochondrial extract was loaded in each lane.  The fold difference between wt and *tor1Δ* normalized to the porin signal is shown on the right.  (**B**) Mitochondrial mass as estimated by the amount of mitochondrial-GFP signal determined by FACS (see Materials and Methods). (**C**) mtDNA copy number determined by real-time PCR (measured as the ratio of the mitochondrial gene target *COX1* relative to the nuclear gene target*    ACT1*).  (**D**) Mitochondrial membrane potential determined by DiOC_6_    staining and FACS analysis.  In B-D means of at least three biological replicates +/- one standard deviation are graphed (** represents a p-value from a student t-test that is <0.01).

The observed increase in mitochondrial translation in *tor1D* strains prompted us to examine additional mitochondrial parameters. Here, we focused on mid-log growth points, where the largest differences in mitochondrial translation and oxygen consumption are observed. First, consistent with the increase in mitochondrial translation, there was an increase in the steady-state levels of mtDNA-encoded OXPHOS subunits (3-12 fold) per mitochondrial mass as judged by western blotting of Cox1p, Cox2p and Cox3p in mitochondrial extracts (Figure 2A). This was accompanied by an increase in the Cox4p OXPHOS subunit (2.2 fold), but not of porin, both of which are encoded by nuclear genes (Figure 2A). This result suggested to us that the OXPHOS machinery was up-regulated more or less specifically and that an overall increase in mitochondrial biogenesis was not occurring. To test this hypothesis, we transformed the strains with a plasmid encoding a mitochondria-targeted GFP protein and measured mitochondrial content by FACS, as well as determined mtDNA copy number, amounts of which usually correlate with mitochondrial abundance. No significant differences in mitochondrial mass (Figure 2B) or mtDNA (Figure 2C) were observed between the wild-type and *tor1D *strains. There also were no obvious differences in mitochondrial distribution or morphology observed by fluorescence microscopy of the GPF-containing strains (data not shown). Altogether, these data indicate that there is an increase in the number of OXPHOS complexes per organelle mass in *tor1D* strains, as opposed to a global up-regulation of the amount of mitochondria per cell. However, despite the fact there is increased mitochondrial OXPHOS components and oxygen consumption, there was a reduction in mitochondrial membrane potential (Figure 2D) and no significant difference in total cellular ATP in *tor1D* strains (data not shown).

To better understand how reduced TOR signaling dynamically effects respiration, we used the TOR kinase inhibitor rapamycin under a variety of conditions. Addition of rapamycin to a wild-type culture from the beginning of growth resulted in a significant and sustained increase in mitochondrial oxygen consumption (Supplementary Figure 1A), similar to that observed in *tor1D *strains. However, rapamycin greatly inhibited the growth rate of these strains (data not shown). In contrast, adding rapamycin at a later point during growth (after the strains reached OD ~1.0) only increased oxygen consumption by ~30% (Supplementary Figure 1B). This increase required the presence of the drug for 2-4 hours, was sustained for at least 30 hours (Supplementary Figure 1B), and depended on both cytoplasmic and mitochondrial translation (i.e. was inhibited by addition of either cycloheximide or chloramphenicol; data not shown).

### Reduced TOR signaling increases the steady-state levels of mitochondrial transcripts

**Figure 3.**
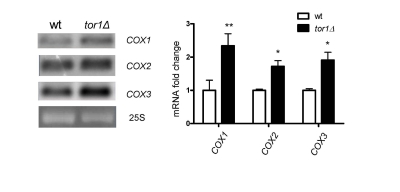
Increase of mitochondrial transcript abundance in tor1Δ strains. Northern analysis of the mtDNA-encoded mRNA transcripts COX1-COX3 from wild-type (wt) and tor1Δ strains is shown, along with ethidium bromide-stained nuclear 25S rRNA as a loading control. Graphed on the right is the mean fold difference in COX1, COX2, and COX3 abundance normalized to 25S rRNA +/- one standard deviation (* designates a p-value <0.05 and ** designates a p-value <0.01 based on a student's t-test).

Given that the overall rates of mitochondrial translation were higher in *tor1D *strains, but mtDNA copy number was not, led us to investigate the whether there were changes in steady-state levels of mitochondrial transcripts that might indicate a mtDNA transcriptional response. Northern blots of three mitochondrial mRNA transcripts revealed that there is a 1.5- to 2.1-fold increase in *tor1Δ* strains (using 25S rRNA as a loading control; Figure 3). Similar changes were observed in the 14S rRNA (data not shown). These data indicate that there is a moderate increase in mitochondrial transcripts in *tor1D *strains, but that this is unlikely to be the primary driving force behind the significantly greater rates of mitochondrial translation and OXPHOS complex abundance observed.

### Global up-regulation of OXPHOS-related proteins in tor1Δ mitochondria revealed by 2D-DIGE 

To gain a better understanding of how reduced TOR signaling affects mitochondria, we have begun to characterize changes in the mitochondrial proteome in *tor1D *strains by two-dimensional, differential gel electrophoresis (2D-DIGE), coupled to mass spectro-metry-based identification of differentially regulated proteins. Given that we observed an increase in OXPHOS subunits/mitochondrial mass by western blot (Figure 2A), we have focused initially on those proteins that were up-regulated by 2-fold or greater in mito-chondria from *tor1Δ* strains (see Materials and Methods). Of the 26 up-regulated spots picked and analyzed based on this 2-fold cutoff, we have unambiguously identified eleven proteins that are at higher steady state-levels in mitochondria purified from *tor1D *strains in the mid-log growth phase (Table 1). In addition to Cox4p, which we had already documented as increased by western blot (Figure 2A), we identified five other OXPHOS components: Cox13p (another subunit of Complex IV), Qcr7 (subunit of Complex III), and Atp2p, Atp5p and Atp7p (subunits of Complex V/ATP synthase). In addition to OXPHOS components, we have thus far identified five other proteins that are up-regulated in mitochondria from *tor1D *strains (Table 1). Three of these (Dld2p, Gcv3p, and Ilv6p) are involved in various aspects of metabolism, one  (Om45p) is an abundant outer mitochondrial membrane protein of unknown function, and the final one (Yhb1p) is involved in nitric oxide detoxification. Altogether, these data solidify our contention that that there is global up-regulation of OXPHOS machinery/organelle in response to reduced TOR signaling, but also indicate that TOR activity impacts mitochondrial proteome composition in other interesting ways. Furthermore, in the case of Gcv1p and Ilv6p, the spots identified of wild-type differ in molecular weight and/or PI from those of *tor1Δ* (data not shown), suggesting that TOR regulates expression and/or processing of these proteins in a unique manner.

### Balanced expression of mitochondrial OXPHOS components is required for extension of chronological lifespan mediated by reduced TOR signaling

**Figure 4.**
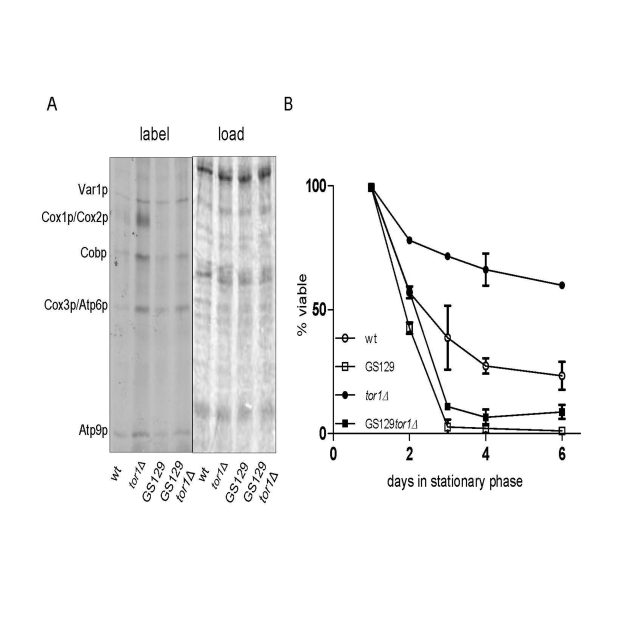
Reduced TOR signaling does not rescue chronological life span in the short-lived GS129 strain with imbalanced mitochondrial translation. **(A)**  Results of mitochondrial translation assays are shown as described in Figure 1.  The strains analyzed are GS122 (wt with regard to RPO41) and GS129 (containing the rpo41-R129D point mutation) in which the TOR1 gene was (tor1Δ) or was not (wt) disrupted (see Materials and Methods). **(B)** Chronological life span curves of the same strains in A. are shown. Three independent colo-nies of each strain were analyzed and the mean % viability +/- one standard deviation is plotted according to the key in the lower left corner.

We previously documented that strains with imbalanced expression of mtDNA-encoded OXPHOS subunits have reduced chronological life span (CLS) [9]. One strain (GS129), in particular, has a severely curtailed CLS due to a point mutation in the amino-terminal domain of mtRNA polymerase (Rpo41p) that results in increased ROS [9]. Given that reduced TOR signaling (due to *TOR1* deletion) increases CLS, in part by increasing the rate of mitochondrial translation [18], we used the GS129 strain background to address the requirement for balanced mtDNA expression in this regard. Deletion of *TOR1* in the GS129 background resulted in an increase in translation of most mtDNA-encoded products to a degree that exceeded that in the isogenic wild-type strain GS122, but less than that observed in the isogenic wild-type *tor1D *strain (GS122 *tor1Δ*) (Figure 4A). However, unlike in the wild-type strain, there was no significant increase in Cox1p translation when *TOR1* was deleted in the GS129 background (Figure 4A). In other words, translation was increased in the GS129 strain in response to reduced TOR signaling, but not in a balanced manner. Analysis of CLS in these strains revealed that deletion of *TOR1* extended life span in the wild-type (GS122) background as expected, but did not significantly increase CLS in the "imbalanced" GS129 strain (Figure 4B). These data indicate that extension of life span by reduced TOR signaling requires balanced up-regulation of OXPHOS components encoded by mtDNA.

### SCH9 is a downstream target of TOR signaling in the regulation of mitochondrial function 

**Figure 5.**
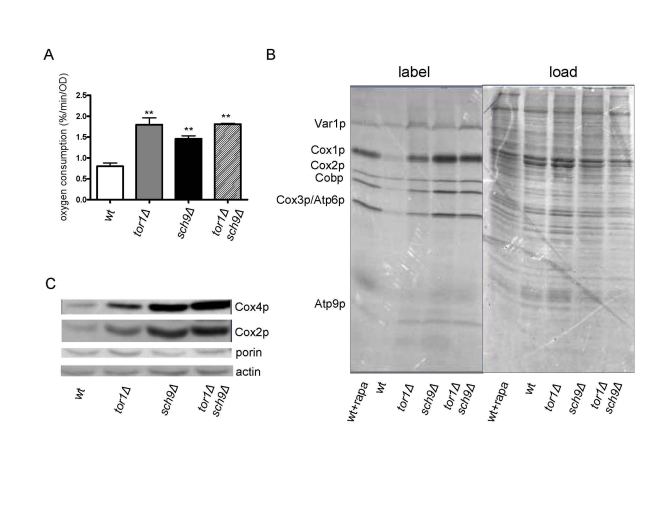
Sch9p mediates TOR-dependent increases in mitochondrial function. Comparative analysisof mitochondria-related parameters in wild-type (wt), *tor1Δ*, *sch9Δ*    and *sch9Δtor1Δ* strains in the DBY2006 genetic background. (**A**)  Mitochondrial oxygen consumption.  (**B**) Mitochondrial translation as described in Figure 1.  (**C**) Western blot of the Cox1p, Cox4p, porin and actin OXPHOS components in the four strains using 100 μg of whole cell extract in each lane.  We use the ratio of porin to actin as one measure of mitochondrial abundance per cell (which is virtually the same between the strains) and the ratio of Cox subunits to porin to demonstrate their specific increase per mitochondrial mass.

Recently, deletion of *SCH9* was also shown to increase expression of mitochondrial OXPHOS genes and mitochondrial respiration [22]. Given that these mitochon-drial phenotypes are similar to those we have documented here and previously in *tor1D *strains, we tested the hypothesis that *SCH9* is downstream of *TOR1* with regard to mitochondrial regulation by simultaneously analyzing isogenic single (*tor1D *or *sch9Δ*) and double (*tor1Δ sch9Δ*) knock-out strains. As reported previously [18], we observed an increase in mitochondrial oxygen consumption in the *sch9Δ* strain that was similar in magnitude (2-fold) to the increase observed in the isogenic *tor1Δ* strain (Figure 5A). However, this increase was not enhanced further in the *tor1Δ sch9Δ* double-mutant strain  (Figure 5A),  consistent  with these  genes being in the same pathway with regard to mitochondrial respiration. Similar results were obtained for mitochondrial translation rates (Figure 5B) and steady-state levels of nucleus and mtDNA-encoded OXPHOS proteins (Figure 5C). However, the *sch9Δ* single mutant had a greater effect than the *tor1Δ* single mutant on these latter three parameters, and there was no synergistic effect observed in the double-mutant strains (Figures 5A and 5B). The fact that the double-mutant strain more closely resembled the *sch9Δ* strain is most consistent with *SCH9* being downstream of *TOR1* in this pathway controlling mitochondrial translation and respiration. This was evidenced further by the fact that addition of rapamycin  to  wild-type strains caused  an increase in mitochondrial translation that was greater in magnitude to that observed in the *tor1Δ*strain (Figure 5B). That is, rapamycin or *SCH9* deletion appears to represent a more complete down-regulation of TOR signaling than deletion of *TOR1*. Finally, comparison of the actin and porin ratio (an indicator of mitochondrial abundance) in the single and double mutant strains (Figure 5C) confirmed that, as was the case for *tor1Δ*, there was no significant increase in overall mitochondrial biogenesis in the *sch9Δ* and *tor1Δ sch9Δ* strains, but rather an increase in the number of OXPHOS complexes per organelle mass, again placing these two genes in the same pathway with regard to mitochondrial function.

**Table 1.** Mitochondrial Proteins Identified as Up-regulated in *tor1Δ* Yeast Strains by 2D-DIGE.

Protein	ID	Protein Function	Expression Ratio *tor1*/wt
**OXPHOS Components**			
Atp2p	gi|151945186	F1F0 ATP synthase beta subunit	2.09
Atp5p	gi|6320504	Subunit 5 of the stator stalk of mitochondrial F1F0 ATP synthase	2.51
Atp7p	gi|151941529	F1F0 ATP synthase subunit d	2.48
Cox13p	gi|6321247	Subunit VIa of cytochrome c oxidase	3.33
Cox4p	gi|6321251	Subunit IV of cytochrome c oxidase	2.19
Qcr7p	gi|6320738	Subunit 7 of the ubiquinol cytochrome-c reductase complex	2.20
**Outer Membrane Protein**			
Om45p	gi|6322055	Protein of unknown function, major constituent of the mitochondrial outer membrane	2.33
**Metabolic Enzymes**			
Dld2p	gi|51830216	D-lactate dehydrogenase, located in the mitochondrial matrix	2.51
Gcv3p*	gi|595540	H-protein subunit of the glycine cleavage system	2.60
Ilv6p*	gi|6319837	Regulatory subunit of acetolactate synthase, which catalyzes the first step of branched-chain amino acid biosynthesis	2.53
**Detoxification Enzyme**			
Yhb1p	gi|6321673	Nitric oxide oxidoreductase, flavohemoglobin involved in nitric oxide detoxification	2.45

### *SCH9* is downstream of *TOR1* in the regulation of chronological life span

**Figure 6.**
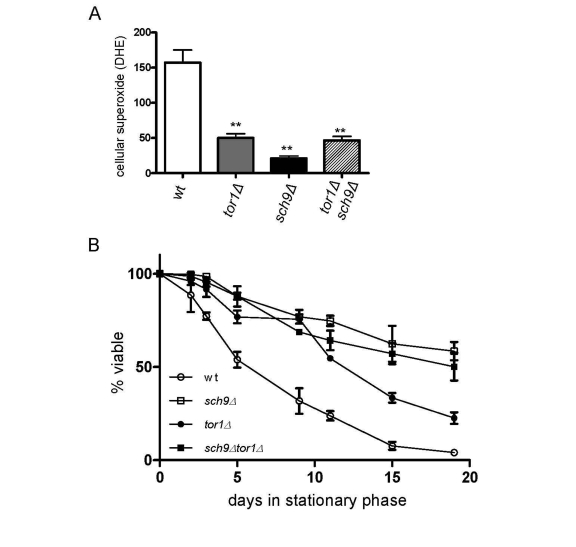
Sch9p is the downstream mediator of TOR-dependent decreases in ROS level and chronological life span extension. Analysis of cellular ROS and chronological life span in the same strains shown in Figure 5.  (**A**) FACS analysis of day 2 stationary phase cells stained for cellular superoxide using dihyroethidium (DHE) is shown.  The mean fluorescence intensity is plotted +/- one standard deviation (** represents a p-value <0.01 according to a student's t-test).  (**B**) Chronological life span plotted as described in Figure 4B.

We previously implicated reduced ROS in stationary phase as a significant factor that increases the CLS of *tor1Δ* strains [18]. A similar reduction in ROS was also observed in *sch9Δ* and *tor1Δ sch9Δ* strains (Figure 6A), again consistent with *SCH9* working in the same genetic pathway as *TOR1* with regard to mitochondria-derived ROS. Finally, as was the case for mitochondrial translation and OXPHOS complex abundance, we found that deletion of *SCH9* increased CLS to a greater degree than deletion of *TOR1*, but that there was no further increase in CLS in the *tor1Δ sch9Δ* double mutant strain (Figure 6B). Altogether, these data solidify the connections between mitochondrial OXPHOS, ROS and CLS and demonstrate that Sch9p is a key downstream factor that mediates the effects of TOR signaling on mitochondrial function and yeast aging.

## Discussion

This study provides significant new insight into the mechanism through which TOR signaling controls mitochondrial function to influence yeast CLS and elucidates which arm of the TORC1 pathway is involved. The primary conclusions we draw from the results obtained are that 1) reduced TORC1 signaling (via deletion of the *TOR1* gene) increases respiration primarily through up-regulation of the number of OXPHOS complexes/organelle, not by increasing overall mitochondrial biogenesis, 2) the up-regulation of OXPHOS complexes involves both mtDNA-encoded and nucleus-encoded subunits and, in terms of mtDNA expression, occurs primarily via translational regulation, 3) in addition to its effects on OXPHOS complex abundance, TOR signaling controls other aspects of mitochondrial proteome dynamics, 4) TOR-dependent changes in mitochondrial function and CLS are mediated by the downstream Sch9p kinase, and 5) it is TOR-dependent alterations of mitochondrial function in the exponential and/or post-diauxic-early stationary growth phases that subsequently impact late stationary-phase survival and extend CLS, which suggests a role of "mitochondrial pre-conditioning" on yeast aging. The basis of these conclusions and additional interpretations are discussed below.

The increase in cellular mitochondrial oxygen consumption (i.e. respiration) in response to reduced TOR signaling reported herein (Figure 5A) and previously [18] could occur by one of several mechanisms that are not mutually exclusive. For example, it could be mediated by direct effects on the activity of existing OXPHOS complexes, by increasing overall mitochondrial biogenesis (resulting in more mitochondria/cell), or by increasing the number of OXPHOS complexes per organelle. Our results demonstrate that increasing organelle OXPHOS complex density is definitely one mechanism at play.

The basis for this conclusion is that, in mitochondrial extracts, we observe increased abundance of both nuclear and mitochondrial OXPHOS subunits, but not other mitochondrial markers (e.g. porin; Figure 2A). This result was substantiated by our initial 2D-DIGE proteomic analysis of highly purified mitochondria from wild-type and *tor1Δ* strains, in which we identified OXPHOS complex subunits (from three separate complexes) as proteins that are in significantly higher abundance in mitochondria from *tor1Δ* cells (Table 1). Finally, there was no increase in overall mitochondrial biogenesis as judged by mtDNA content (Figure 2C), labeling of mitochondria with a GFP marker and analyzing them by FACS (Figure 2B), and western blot comparisons of mitochondrial and cytoplasmic markers (Figure 5C). While increased OXPHOS complex density is clearly occurring, we have not eliminated the possibility that there are also TOR-dependent effects on the enzymatic activity of the complexes that contribute to the increase in oxygen consumption.

Our 2D-DIGE results are not entirely consistent with a recent proteomic study of rapamycin-treated yeast cell [23], where fewer OXPHOS proteins were identified as up-regulated. We found that addition of rapamycin during the growth phase impacts mitochondrial oxygen consumption on a longer time scale and to a lesser degree compared to adding rapamycin from the beginning of the growth experiment (i.e. at inoculation; Supplementary Figure 1). The latter condition is in some ways more similar to the *tor1Δ* strains analyzed in this study in that, in this case, TOR signaling is reduced throughout all stages of growth. Thus, differences in the timing and/or degree of TOR inhibition may explain the different results obtained in the two studies.

To increase OXPHOS complex density as a means to increase mitochondrial oxygen consumption is to our knowledge a unique mechanism of mitochondrial regulation levied by the TOR pathway. We originally hypothesized that this would lead to greater mitochondrial membrane potential due to the increase in electron transport activity and perhaps also a higher cellular ATP. However, this was not the case; there was instead a decrease in membrane potential in *tor1Δ* strains (Figure 2D) and no change in cellular ATP (data not shown). Thus, in *tor1Δ* strains, there in an increase in electron transport activity (i.e. oxygen consumption) and a decrease in mitochondrial membrane potential, which equates to a mitochondrial network with overall lower energy capacity on average. One potential explanation for this result is that reduced TOR signaling is leading to an increase in uncoupled respiration. This would lead to increased oxygen consumption in an attempt to maintain the membrane potential in the face of the proton leak and an inability to simultaneously increase ATP production. Since mild uncoupling also increases CLS [11], this indeed may prove to be the mechanism through which TOR signaling influences aging in yeast. Testing this hypothesis is a logical area of future investigation, but certainly other explanations can be envisioned.

In order to affect an increase in OXPHOS complexes per mitochondrion, the cell needs to increase the production and/or stability of both mtDNA-encoded and nucleus-encoded OXPHOS subunits, while not inducing a full mitochondrial biogenesis response. How reduced TOR signaling accomplishes this remains to be determined, yet several insights are gleaned from our results. First, we observe an increase in both mtDNA-encoded and nucleus-encoded OXPHOS subunits (Figure 2A, Table 1), thus TOR signaling is affecting both mitochondrial and nuclear gene expression simultaneously. According to our results, this is occurring both at the mRNA level (Figure 3) and at the translational level (Figure 1) in mitochondria, but not at the level of protein stability to any obvious degree (Figure 5B, Supplementary Figure 2). Since transcript-tion and translation are coupled in mitochondria [24-26], these changes probably work together to mediate the increase in OXPHOS complex abundance in *tor1Δ* strains. Although, the translational control appears to contribute to a greater extent, given only modest changes in mitochondrial transcript levels are observed. However, the change in mitochondrial transcripts of *tor1Δ* strains might represent a reduction of glucose repression, which is known to induce mitochondrial transcription [27,28] and mimic the effects of *tor1Δ* on respiration and CLS based on our previous study [18]. Interesting in this regard is the key role of the Snf1p kinase in the glucose repression phenomenon [29]. Snf1p is the yeast ortholog of mammalian AMP kinase, which negatively regulates mTOR signaling in response to energy charge by activating Tsc2, an inhibitor of mTORC1 [30]. Though a Tsc2 ortholog appears to be absent in yeast, these correlations might suggest an evolutionarily conserved regulatory framework that links glucose metabolism, TOR signaling, mitochondrial gene expression and life span.

Whether the increase in nuclear OXPHOS gene expression is mediated at the transcriptional or post-transcriptional level remains to be determined, as does the identity of the putative TOR-regulated mitochondrial factors that meditate the increase in mitochondrial mRNA transcription/stability and translation of mtDNA-encoded OXPHOS subunits. Certainly, nuclear transcription factors that are known to be downstream of TORC1 [31], involved in nuclear-mitochondrial signaling [32], or in glucose repression of mitochondrial function [33] are obvious candidates to test with regard to the nuclear gene expression response. And, with regard to TOR-dependent factors that regulate mitochondrial gene expression directly, the mitochondrial transcription machinery, mitochondrial ribosomes, or the various general and specific translational activators [5] are likely candidates to consider in future studies. Furthermore, since our results clearly implicate Sch9p as the key mediator of the TORC1-mitochondria-CLS pathway (Figure 6B), searching for mitochondrial substrates of Sch9p as potential downstream targets that execute changes in mitochondrial gene expression and OXPHOS activity would likely be fruitful.

The fact that up-regulation of mitochondrial oxygen consumption [18] and mitochondrial translation (Figure 1) in *tor1Δ* strains occurs only in log-phase and early stationary phase cultures (and not later in stationary phase) strongly suggests that TOR-dependent mitochondrial changes that occur early are responsible for the life span extension later in stationary phase. The concept of early mitochondrial-related events effecting life span has been promoted by others in aging studies in *C. elegans* [34,35] and is also consistent with the observation of Piper and colleagues that previous conditioning of yeast to respiratory conditions extends CLS in subsequent cultures [36]. While, at this point, the molecular explanation for this "mitochondrial pre-conditioning" effect is not clear, we consider ROS signaling as one potential model. This idea is attractive because the rate of production of ROS from the mitochondrial electron transport chain is likely an accurate reflection of mitochondrial OXPHOS activity and/or redox status that could be used by cells as a retrograde signal to modulate nutrient-sensing pathways. Although we have not observed a significant change in the steady-state level of superoxide in log-phase *tor1Δ* cells (data not shown), it is possible that other ROS species may be relevant or that the steady-state measurements are not accurately predicting the rate of mitochondrial ROS production. Alternatively, we observed up-regulation of Yhb1, a nitric oxide detoxifying enzyme in *tor1Δ* mitochondria, but not Sod2p (data not shown; [18]). These results might suggest a role for NO and/or other reactive nitrogen species as relevant. Interesting in this regard, as is the case in *tor1Δ* cells (Table 1), Yhb1p localizes to mitochondria under anaerobic condition [37]. This, coupled to our observation that hypoxic conditions bypass the extension of CLS by *TOR1* deletion [18] might suggest that reduced TOR signaling and anaerobic conditions share a common route to impact life span that may involve NO metabolism. Future studies along these and related lines, as well as further characterization of TOR-dependent changes in the mitochondrial proteome should be most revealing in terms of understanding how the TOR-mitochondria axis controls aging and deciphering the complex relationships between OXPHOS activity, ROS (and/or other reactive species), nutrient sensing, and life span. This, in turn, may provide new inroads into understanding and perhaps counteracting age-related pathology in humans.

## Materials and methods

Yeast strains. Unless otherwise stated, strains of the DBY2006 (*MATa his3-Δ200 leu2-3,-112 ura3-52 trp1- Δ1 ade2-1*) background were used exclusively. The GS122 and GS129 strains are derivatives of DBY2006 that have plasmid-borne *RPO41* and *rpo41-R129D* alleles covering a chromosomal disruption of the endogenous *RPO41* gene and have been described previously [24]. These strains were used for the experiments presented in Figures 1 and 4. The *TOR1* gene was disrupted in these strains as described previously [18]. The *SCH9* gene was disrupted using a standard *HIS3* knockout cassette [38]. Briefly, the *HIS3* in pRS313 was PCR amplified with primers ACCACCGCTATTAGTCAGGACTTATATGCAATGGGCACAACAGGAATAACAAGATTGTACTGAGAGTGCAC (*SCH9*_LeftDel) and CATCATTGATGTCC TCGTCCCCGTCATCATCGATGACATCTTCGTCTG GACTGTGCGGTATTTCACACCG (SCH9_RightDel).

Gel-purified amplicons were used to transform wild-type and *tor1Δ* DBY2006. His+ transformants were selected on his- plates and single colonies were picked and verified by PCR. The mitochondrial GFP expressing yeast strains were generated by transforming wild-type DBY2006 and *tor1Δ* with pYX142-SU9-GFP [39].

Mitochondria purification. Mitochondria were isolated from yeast (from cultures grown to OD_600_=1.0 in selective media) by differential centrifugation followed by sucrose-gradient fractionation as described [40]. For 2D-DIGE, the purity of mitochondrial preparations was checked by western blot analysis with anti-actin (Chemicon, 1:1000), anti-alkaline phosphatase (Molecular Probe 1:1000), anti-Dol-P-Man synthase (Molecular Probes, 1:1000) antibodies to control for contamination of cytoplasm, vacuolar membrane, and ER membrane respectively. Only residual ER contamination was present in the purified mitochondrial preparations (data not shown).

Mitochondrial translation assay. Unless otherwise stated, mitochondrial translation assays were performed as described [25], except the following: all reactions were carried out at 30^0^C, gradient gels (6-20%, Figures 1 and 4; 15-22.5%, Figure 5) were used to resolve translation products and electrophoresis was conducted at a constant current of 30 mA.

Chronological Life Span Assay. Chronological life span was assayed as described previously [9,18] Unless otherwise stated, viability was determined by staining with 0.4% trypan blue.

Measurement of mtDNA Copy Number. The mtDNA copy number was determined using a quantitative real-time PCR procedure as described previously [41,42].

Northern Analysis. Northern blots were performed as described previously [24,42]. Briefly, 5 μg of total RNA extracted from yeast (cultured to an OD_600_=1) was separated on a 1.5% agarose-formaldehyde gel and then transferred to a nylon membrane by capillary action. Radiolabeled probes were synthesized by PCR with ^32^P-dCTP an added to the membranes in rapid-hyb buffer (GE Healthcare) and incubated overnight at 42 ^0^C. The membrane was washed at room temperature with increasing stringency before visualization by auto-radiography as described in the references cited above.

Western Blot Analysis. Western blots of mitochondria and total cell extracts (from cultures at (OD_600_=1) was performed as described previously [18,25]. Proteins were separated on a 10% SDS-PAGE, transferred to a PVDF membrane, and incubated with the indicated primary and HRP-conjugated anti-mouse secondary (Molecular Probes) antibodies as described previously [18, 25]. Anti-Cox4p (MitoSciences) antibody (not used previously) was diluted 1:1000 for incubation with the blocked membrane.

2D-DIGE. Mitochondrial extraction followed steps mentioned in the "mitochondrial extraction" section. 2D-DIGE was conducted by the W.M. Keck Facility of Yale University (http://keck.med.yale.edu/dige/). Briefly, protein samples were prepared by TCA-precipitation of mitochondrial extracts from DBY2006 and *tor1Δ*. The samples were further cleaned with 2-D Clean-Up Kit (GE Healthcare) and labeled with CyDye DIGE fluors (Amersham). 50 μg of the labeled samples were resolved on a 2D gel (Ettan DIGE system from Amersham). A representative 2D gel and the distribution of up-regulated and down-regulated proteins is shown in Supplementary Figure 3. In this study, 26 proteins that were up-regulated by 2-fold or more were selected for MALDI-MS/MS analysis. We were able to unambiguously identify 11 of these based on multiple peptide matches.

Flow Cytometry. All analysis was performed on a Beckton-Dickenson FACSCalibur.  Analysis of yeast ROS using DHE was performed as described previously [9]. For measurement of mitochondrial potential, cells from a growing culture were pelleted by centrifugation and washed with phosphate-buffered saline (PBS). DiOC_6 _(Molecular Probes) was diluted to a final concentration of 200 nM in PBS and used to resuspend the cells. The cell suspension was then incubated for 30 minutes at 30 ^0^C, washed twice with PBS, and analyzed by flow cytometry using the FL3 channel without compensation. For measurement of mitochondrial mass, cultured GFP expressing yeast cells were pelleted, washed once and then re-suspended in PBS, and subject to flow cytometry analysis using the FL1 channel without compensation.

## Supplementary figures

Supplementary Figure 1 Rapamycin affects mitochondrial oxygen consumption differently depending on whether it is added at the beginning of the culture or during exponential growth. **(A)** Mitochondrial oxygen consumption of a wild-type (DBY2006) culture supplemented with 200 nM rapamycin upon inoculation. Indicated on the x-axis are the time after inoculation and the OD600 at that time point. **(B)** Same as in **(A)** except rapamycin was added during active growth (OD600 of 1) and the times indicate the time after addition and the OD600 at that time point

Supplementary Figure 2 Newly synthesized mtDNA-encoded OXPHOS subunits have similar stability in wild-type and tor1Δ strains. The experiment shown is identical to that described in Figure 5B, except non-radioactive amino acids were added to the culture (cold chase) at 30°C for 90 minutes, instead of 10 minutes.

Supplementary Figure 3 2D-DIGE analysis of changes in the mitochondrial proteome in tor1Δ strains. (**A**) 2D gel image of tor1Δ mitochondrial proteins. Wild-type (DBY2006) and tor1Δ were labeled with cy3 and cy5, respectively. The indicated pI (x-axis) and molecular weight (y-axis) are approximate.

Supplementary Figure 3B (**B**) Spot distribution of differentially expressed mitochondrial proteins. The x-axis indicates the cy5/cy3 ratio (positive values indicate up-regulation in tor1Δ strains; negative values down-regulation). The left y-axis shows spot frequency; the right y-axis represents the maximum spot volume of a given spot (pair). Frequency distribution of the log volume ratios (rough curve) is plotted, while the normalized model frequency (smooth curve) was fitted to the spot ratios so that the modal peak is zero. Vertical lines indicate a 1.5-fold difference cutoff in cy5/cy3 spot volume ratio.
