# Supplementary figures and images for: Extension of chronological life span by reduced TOR signaling requires down-regulation of Sch9p and involves increased mitochondrial OXPHOS complex density
Source: Aging (Albany NY). 2009 Jan 28;1(1):131–45. doi: 10.18632/aging.100016 (PMC2815770; doi:10.18632/aging.100016)

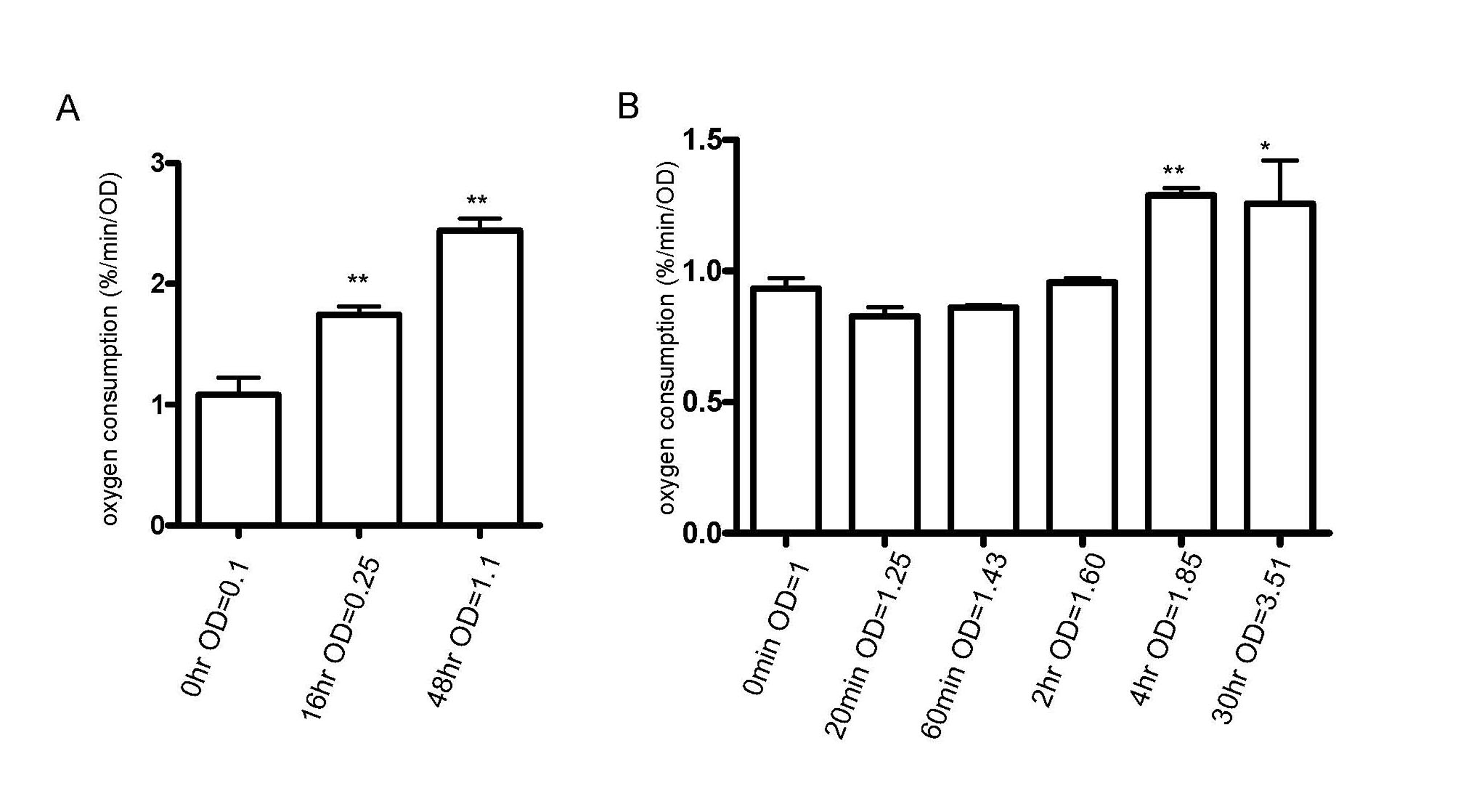

Supplement: Supplementary Figure 1 — (A) Mitochondrial oxygen consumption of a wild-type (DBY2006) culture supplemented with 200 nM rapamycin upon inoculation. Indicated on the x-axis are the time after inoculation and the OD600 at that time point. (B) Same as in (A) except rapamycin was added during active growth (OD600 of 1) and the times indicate the time after addition and the OD600 at that time point [file aging-01-131-s001.tif]

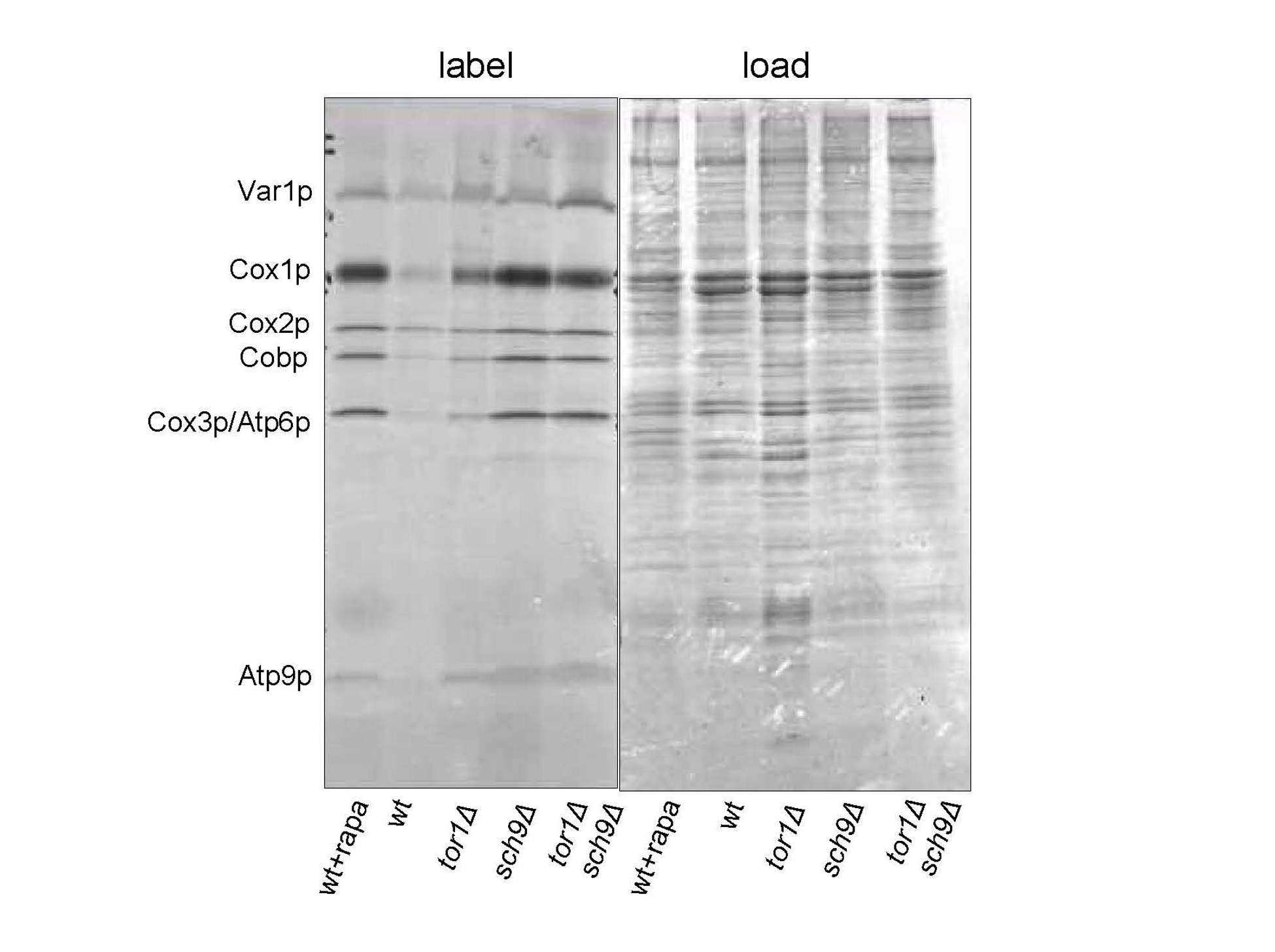

Supplement: Supplementary Figure 2 — The experiment shown is identical to that described in Figure 5B, except non-radioactive amino acids were added to the culture (cold chase) at 30°C for 90 minutes, instead of 10 minutes. [file aging-01-131-s002.tif]

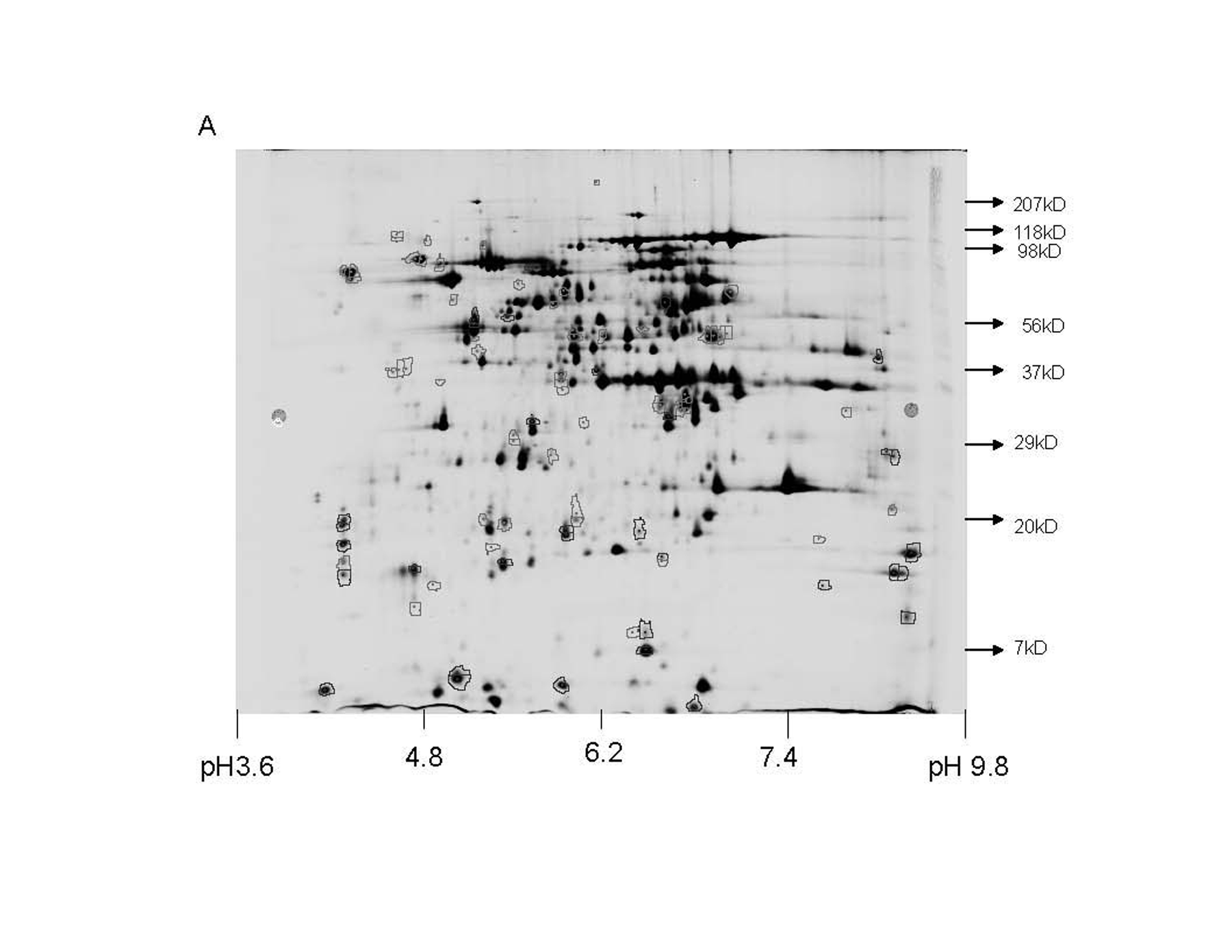

Supplement: Supplementary Figure 3 — (A) 2D gel image of tor1Δ mitochondrial proteins. Wild-type (DBY2006) and tor1Δ were labeled with cy3 and cy5, respectively. The indicated pI (x-axis) and molecular weight (y-axis) are approximate. [file aging-01-131-s003.tif]

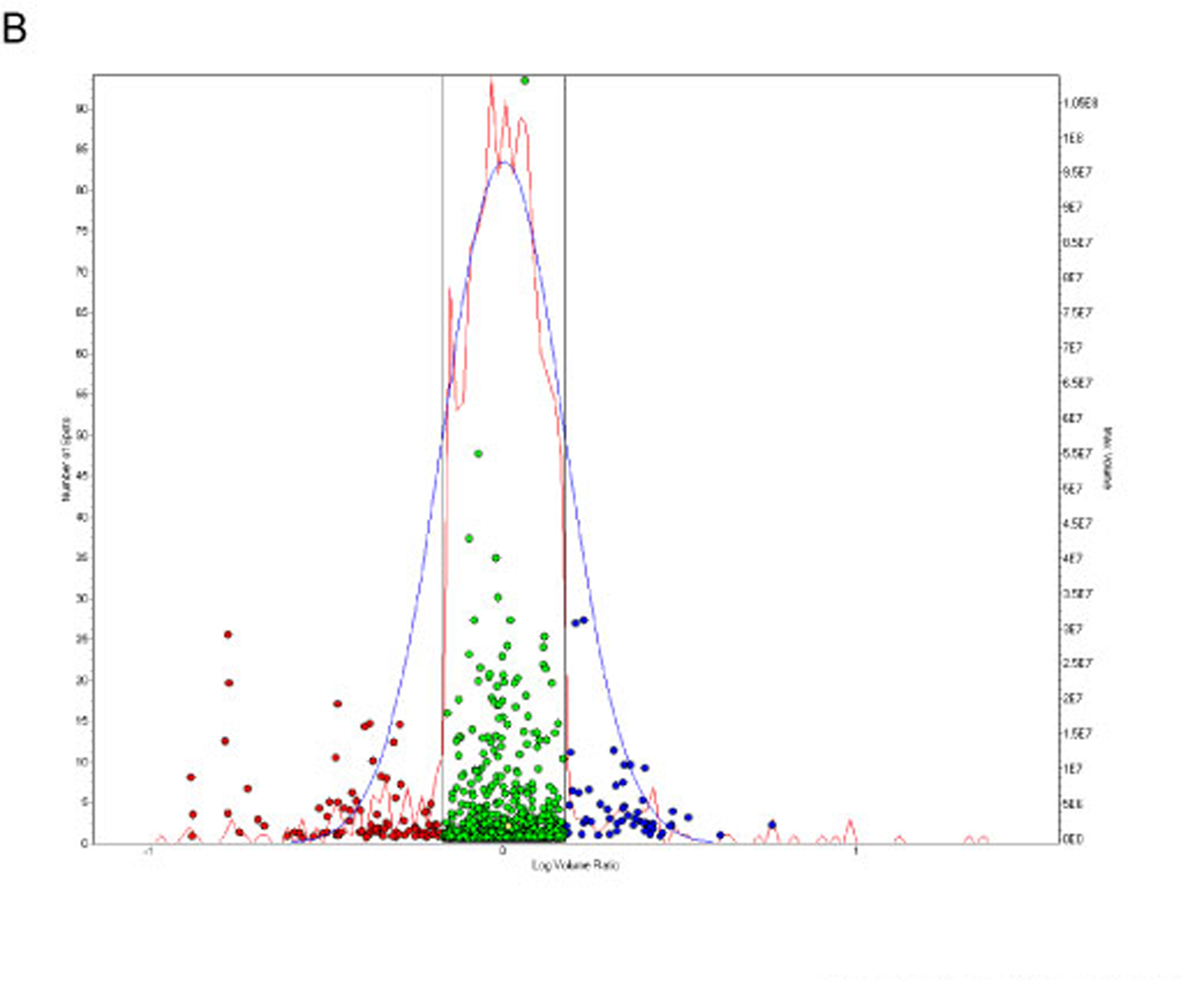

Supplement: Supplementary Figure 3B — (B) Spot distribution of differentially expressed mitochondrial proteins. The x-axis indicates the cy5/cy3 ratio (positive values indicate up-regulation in tor1Δ strains; negative values down-regulation). The left y-axis shows spot frequency; the right y-axis represents the maximum spot volume of a given spot (pair). Frequency distribution of the log volume ratios (rough curve) is plotted, while the normalized model frequency (smooth curve) was fitted to the spot ratios so that the modal peak is zero. Vertical lines indicate a 1.5-fold difference cutoff in cy5/cy3 spot volume ratio. [file aging-01-131-s003B.tif]
